# Sleep Duration and Excessive Daytime Sleepiness Are Associated with Obesity Independent of Diet and Physical Activity

**DOI:** 10.3390/nu10091219

**Published:** 2018-09-03

**Authors:** Andrea Maugeri, Jose R. Medina-Inojosa, Sarka Kunzova, Antonella Agodi, Martina Barchitta, Ondrej Sochor, Francisco Lopez-Jimenez, Yonas E. Geda, Manlio Vinciguerra

**Affiliations:** 1 International Clinical Research Center, St Anne’s University Hospital, 65691 Brno, Czech Republic; andreamaugeri88@gmail.com (A.M.); sarka.kunzova@fnusa.cz (S.K.); ondrej.sochor@fnusa.cz (O.S.); 2 Department of Medical and Surgical Sciences and Advanced Technologies “GF Ingrassia”, University of Catania, 95123 Catania, Italy; agodia@unict.it (A.A.); martina.barchitta@unict.it (M.B.); 3 Department of Cardiovascular Medicine, Division of Preventive Cardiology, Mayo Clinic, Rochester, MN 55905, USA; MedinaInojosa.Jose@mayo.edu (J.R.M.-I.); lopez@mayo.edu (F.L.-J.); 4 Translational Neuroscience and Aging Program, Mayo Clinic, Scottsdale, AZ 85259, USA; geda.yonas@mayo.edu; 5 Division of Epidemiology, Department of Health Sciences Research, Mayo Clinic, Rochester, MN 55905, USA; 6 Department of Psychiatry and Psychology, Mayo Clinic, Scottsdale, AZ 85259, USA; 7 Department of Neurology, Mayo Clinic, Scottsdale, AZ 85259, USA

**Keywords:** diet, physical activity, overweight, central obesity, body mass index

## Abstract

In the European Union, Czech Republic ranks 3rd and 6th for the incidence of obesity and cardiovascular diseases, respectively. Worldwide, short sleep duration and excessive daytime sleepiness (EDS) characterize obese subjects, which in turn exhibit scarce physical activity and unhealthy diet. We aimed to understand the relationship between irregular sleep patterns, obesity and lifestyle factors, such as diet and physical activity, in a vulnerable Czech population. 1482 members of the Kardiovize cohort, a random sample of the Czech urban population, were included in a cross-sectional study. Exposure variables included self-reported sleep duration and EDS, assessed by the Epworth Sleepiness Scale. Primary outcomes were BMI and waist-to-hip ratio or prevalence of obesity and central obesity. Covariates included physical activity and diet. Associations and interactions between variables were evaluated using logistic regression analyses. After adjustment for covariates, short sleep duration (<7 h) was associated with greater odds of overweight (BMI > 25; OR = 1.42; 95%CI = 1.06–1.90; *p* = 0.020) and obesity (BMI > 30; OR = 1.40; 95%CI = 1.02–1.94; *p* = 0.047), while EDS was associated with greater odds of central obesity (OR = 1.72; 95%CI = 1.06–2.79; *p* = 0.030), independent of diet and physical activity. However, due to the cross-sectional nature of our study, further prospective, large-scale studies are needed to evaluate the etiological link and causality between sleep disturbances and obesity.

## 1. Introduction

Sleep homeostasis is a phenomenon by which organisms compensate for sleep loss or surplus via homeostatic and circadian mechanisms to maintain a constant physiology. The ubiquity of sleep homeostasis across many animal species, including humans, suggests that it underscores essential functions [1]. Indeed, irregular sleep patterns or sleep disruptions can have adverse long-term health consequences, including hypertension, dyslipidaemia, cardiovascular disease (CVD), metabolic syndrome, type 2 diabetes and obesity [2]. The latter, defined as a body mass index (BMI) ≥ 30, is associated with increased total and cardiovascular mortality [3,4] and affects one third of the adult population worldwide [5]. Consequently, there is need for global action to tackle the obesity pandemic. Body fat can accumulate in specific tissues and thus be unevenly distributed throughout the body. Central obesity, also known as abdominal or visceral obesity, is the presence of excess abdominal fat around the stomach and abdomen. Central obesity is measured by specific indices, such as waist-to-hip ratio (WHR ≥ 0.85 in women and WHR ≥ 0.90 in men) and is not confined only to those with a high BMI or who are obese but can be present even in those with normal overall body weight. Affected individuals with normal weight central obesity (NWCO) have a greater total mortality risk than those of normal weight, overweight or who are obese but without central obesity [6]. An association between short sleep duration and obesity across all age groups has been demonstrated in several epidemiological studies and meta-analyses [7,8,9]. Notably, the most recent meta-analysis by Wu et al. indicated that short sleep but not long sleep duration, was significantly associated with incidence of obesity. However, the authors concluded that, considering the relatively small number of studies, further research is needed to confirm these findings [9]. Similar to sleep duration, excessive daytime sleepiness (EDS)—characterized by persistent sleepiness and general lack of energy despite apparently adequate or even prolonged night-time sleep—seems to be associated with obesity independent of night sleep patterns [10]. Sleep loss affects hypothalamic function and the communication network between the brain and the immune system through sympathetic overstimulation, hormonal imbalance and inflammation, generating cravings and lipogenesis. These pathways might explain the association between sleep loss and excessive daytime sleepiness and obesity [11]. The pathogenesis of obesity is also affected by lifestyle factors including physical activity, sedentary behaviours and dietary habits and genetic risk or susceptibility [12]. Although several studies investigated the association of sleep characteristics with obesity, adjusting for the effect of lifestyle factors [13,14,15,16], how sleep duration and EDS interact with lifestyle factors (i.e., diet and physical activity) to mediate risk of obesity and central obesity remains unclear.

We recently established the Kardiovize Brno 2030 study to investigate the complex relationships between cardiovascular diseases and a range of biological, psychosocial, environmental and behavioural risk factors. We prospectively recruited a cohort of ~1% randomly-selected residents in an urban population of Brno, the second largest city of the Czech Republic [17]. Previous analyses on the Kardiovize cohort suggested that dietary patterns, eating timing and physical activity are associated with indices of obesity and other cardio-metabolic risk factors [18,19,20]. However, to our knowledge, no studies investigated the relationship and the interaction between sleep characteristics, lifestyle and obesity in the Czech population and more in general Central European populations. Among the 28 state members of European Union, Czech people are particularly vulnerable to obesity and to CVD, ranking 3rd for the incidence of obesity [21] and 6^th^ for number of deaths due to CVD (ischemic heart diseases and stroke) [22]. Thus, uncovering the main risk factors of obesity and their interactions should be a matter of further and more detailed investigation. Here, our cross-sectional analysis examined the association between self-reported sleep duration and EDS with indices of obesity in the Kardiovize Brno 2030 cohort, taking into account potential interactions with lifestyle risk factors.

## 2. Materials and Methods

### 2.1. Study Design

Kardiovize Brno 2030 is a prospective cohort study enrolling a random sample of 25–64-year-old residents of the city of Brno, Czech Republic [17]. Recruitment and baseline examinations were completed in 2014 with planned follow-up at 5-year intervals through 2030. The baseline study protocol was approved by the ethics committee of St Anne’s University Hospital, Brno, Czech Republic (reference 2 G/2012), in accordance with the Declaration of Helsinki. Data were stored using the web-based research electronic data capture (REDCap) [23]. For the current analysis, we used data from participants with complete anthropometric measurements, sociodemographic and life-style information. To avoid potential confounding, we excluded subjects with previous/current cardiovascular disease, obstructive sleep apnoea, hypothyroidism and hyperthyroidism, depression and night- and shift-workers.

### 2.2. Sleep Measures

Self-reported sleep duration on weekdays was obtained from answers to the following question: “How many hours do you sleep on average during a 24-h period?” The response categories ranged from “≤5 h” to “≥11 h” at intervals of one hour. Sleep duration was classified as short (<7 h), normal (7–9 h) or long (>9 h), based on the joint consensus statement of the American Academy of Sleep Medicine and Sleep Research Society [24]. Daytime sleepiness was evaluated using the Epworth Sleepiness Scale (ESS) previously translated and validated in Czech [25]. ESS results range from 0–24 with high scores reflecting high levels of sleepiness and EDS was defined as an ESS ≥10 [26,27]. In addition, participants were classified by the risk of having sleep apnoea using the Berlin Questionnaire, which consist of three categories related to risks and symptoms of sleep disorders (i.e., snoring, stop breathing, tiredness and fatigue, falling asleep and high blood pressure and/or BMI). As described elsewhere [28], participants were classified at high risk of sleep apnoea if they reported two or more categories with a positive score.

### 2.3. Anthropometric Assessment

Anthropometric measurements were performed in the morning by trained professionals, according to previously described protocols [17]. Height and weight were measured using a medical digital scale with meter (SECA 799; SECA, GmbH and Co. KG, Hamburg, Germany) to the nearest 1 cm and 1 kg. BMI was calculated as weight in kilograms divided by height in meters squared and analysed as a continuous or categorical variables as follows: underweight (<18.5 kg/m^2^), normal weight (18.6–24.9 kg/m^2^), overweight (25–29.9 kg/m^2^) and obese (≥30 kg/m^2^). Waist, hip and neck circumferences were determined to the nearest 1 cm by manual tape measurement. Waist-to-hip ratio (WHR) was calculated dividing the waist circumference by hip circumference. Central obesity was defined as WHR > 0.90 for men and > 0.85 for women [29]. Body fat mass (BFM) was assessed by direct segmental multi-frequency bioelectrical impedance analysis (InBody 370; BIOSPACE Co., Ltd., Seoul, Korea).

### 2.4. Lifestyle Factors

Physical activity levels and intensity (walking, moderate and vigorous) were assessed across four domains (leisure time, work/commuting, home and garden/yard), using the long version of the International Physical Activity Questionnaire (IPAQ-L) [30] translated into Czech. Physical activity was reported as Metabolic Equivalent of Task (MET-min/week) and classified as: (i) high activity (vigorous-intensity activity on ≥3 days and accumulating ≥1500 MET-min/week or ≥7 days of any combination of walking, moderate-intensity or vigorous intensity activities achieving a minimum of ≥3000 MET-min/week); (ii) moderate activity (≥3 days of vigorous activity of ≥20 min per day or ≥5 days of moderate-intensity activity or walking of ≥30 min per day or ≥5 days of any combination of walking, moderate-intensity or vigorous intensity activities achieving ≥600 MET-min/week); (iii) low activity (subjects who not meet criteria for categories (i) or (ii)) [30].

Dietary data were collected by a 43-item Food Frequency Questionnaire (FFQ) as described elsewhere [18] and diet was assessed based on the following components: ≥4.5 cups/day of fruits and vegetables (approximated as at least 4.5 servings/day); ≥3.5 oz servings/week of fish (approximated as at least two 3–5 oz. servings/week); ≥3.1 oz servings/day of whole grains (approximated as at least three servings/day); sodium (<1500 mg/day); and ≤36 oz/week of sugar sweetened beverages (approximated as a maximum of four glasses/week). Diet was categorized according to the achievement of 0–1, 2–3 or 4–5 of these components [31].

For smoking, subjects were classified as either non-smokers (never smoked or not smoked for >12 months) or current smokers (daily or occasionally).

### 2.5. Other Covariates

Face-to-face health interviews were carried out to assess demographic/socioeconomic status and personal history of disease. The following information were recorded: (i) demographics and socioeconomic status (age, gender, educational level and employment status); ii) self-reported medical history (diagnosis and treatment of hypertension, hyperlipidaemia and diabetes). Hypertension was defined as blood pressure ≥140/90 mmHg, a prior diagnosis of hypertension or taking antihypertensive drugs. Hyperlipidaemia was defined as either total cholesterol ≥5.0 mmol/L, LDL cholesterol ≥3 mmoL/L, triglycerides ≥1.7 mmoL/L, or taking lipid-lowering drugs. Diabetes mellitus was defined as a prior diagnosis of diabetes mellitus, a fasting glucose ≥7 mmoL/L, or taking antidiabetic drugs.

### 2.6. Statistical Analyses

Descriptive statistics were used to characterize the study population, using frequency, mean and standard deviation (SD), or median and interquartile range (IQR). Prior to analysis, the normal distribution of all variables was checked using the Kolmogorov-Smirnov test. Continuous variables underlying skewed distribution were compared using nonparametric methods (i.e., Mann-Whitney U test for comparisons between two groups or the Kruskal–Wallis test for comparisons between three or more groups). Categorical variables were compared using Chi-square test. Associations of short sleep duration and EDS with indices of obesity (overweight-obesity: BMI ≥ 25.0; obesity: BMI ≥ 30.0; central obesity: WHR > 0.9 for men and > 0.85 for women) were investigated using binary logistic regression, with normal sleep duration and no EDS used as reference. Data are reported as odds ratio (OR) and the corresponding 95% confidence intervals (95%CI). The small number of long sleepers (>9 h) avoided to assess the association of long sleep duration with indices of obesity.

To assess how the relationship of short sleep duration and EDS with obesity might be modulated by lifestyle factors (diet and physical activity), we ran two regression models that included an increasing number of covariates: Model 1 included age, sex, neck circumference, smoking status, marital status, employment, high risk of sleep apnoea, medical history and treatments of diabetes, hypertension and hyperlipidaemia; Model 2 included all variables in Model 1 but also adjusted for diet, total energy intake and physical activity. We also investigated the interactions of sleep duration with EDS, diet and physical activity on BMI and WHR using a general linear model. Tests for interaction were fitted by treating all independent variables as categorical and BMI and WHR as continuous. All statistical tests were two-sided and *p* values < 0.05 were considered statistically significant. All statistical analyses were conducted using SPSS software (version 22.0, SPSS, Chicago, IL, USA).

## 3. Results

### 3.1. Study Population

From a total of 2160 Kardiovize participants, current analysis was conducted on those with complete health interview and anthropometric assessment, which satisfied inclusion/exclusion criteria. Particularly, we excluded 100 subjects with CVD, 193 with hypo- or hyperthyroidism, 11 with obstructive sleep apnoea and 280 night– or shift–workers; the remaining 94 subjects were excluded due to incomplete anthropometric measurements, sociodemographic and/or life-style information. Accordingly, a total of 1482 participants, aged 25 to 65 years (mean = 45.9 years; SD = 11.2), were included: 50.5% were female and 18.9% were current smokers. While only 3.9% regularly consumed at least four healthy dietary components, more than half (55.1%) were highly physically active. The prevalence of hypertension, hyperlipidaemia and diabetes mellitus was 37.5%, 66.7% and 7.7%, respectively and 41% were overweight or obese, with 16.3% and 40.4% meeting or exceeding the cut-off for obesity and central obesity, respectively.

### 3.2. Main Characteristics and Indices of Obesity by Sleep Duration

We first assessed the main characteristics of participants by categories of sleep duration (Table 1). While the majority subjects exhibited normal sleep length (7–9 h; 68.2%), ~30% had short sleep duration (<7 h/night) and 0.9% reported long sleep duration (>9 h/night). The age of participants increased with decreasing sleep duration (*p* < 0.001). Physical activity, expressed as MET-min/week, decreased with increasing sleep duration (*p* < 0.001) but the distribution of physical activity categories did not differ. By contrast, differences in the use of anti-diabetics, lipid lowering medications and/or antihypertensive drugs across categories of sleep duration were not evident. Notably, the proportion of participants at high risk of sleep apnoea decreased with increasing sleep duration (*p* < 0.001).

We then interrogated indices of obesity by categories of sleep duration (Table 2). In general, we observed that indices of obesity decreased with increasing sleep duration. Compared to short duration sleepers, both BMI and prevalence of obesity were lower among normal and long sleepers (*p* < 0.001 and *p* = 0.002, respectively). In addition, we observed significant differences in WHR (*p* = 0.018) and body fat mass (*p* = 0.004), with a lower prevalence of central obesity among normal and long sleepers (*p* = 0.032).

### 3.3. Main Characteristics and Indices of Obesity by Excessive Daytime Sleepiness

Next, we analysed the main characteristics of the 11.0% participants with EDS compared with those who did not report EDS (Table 3). Here, the proportion of males and the total energy intake was higher in those who reported EDS compared to those without EDS (*p* = 0.036 and *p* = 0.002, respectively). No significant differences were found for any of the other socio-demographic and lifestyle factors studied. However, we noted a high proportion of short sleepers and/or participants at high risk of sleep apnoea in those who reported EDS compared to those without EDS (*p* < 0.001 and *p* = 0.026, respectively).

We also observed that those who reported EDS had a higher BMI and WHR compared to those who did not report EDS (*p* = 0.020 and *p* = 0.013) (Table 4). Although no significant difference was observed in the distribution of overweight and or obesity, the prevalence of central obesity was significantly higher in the EDS group (*p* = 0.001). In addition, subjects with EDS had a higher neck circumference compared to those who did not report EDS (*p* = 0.007).

### 3.4. Association of Sleep Duration with Indices of Obesity

**Figure 1:**
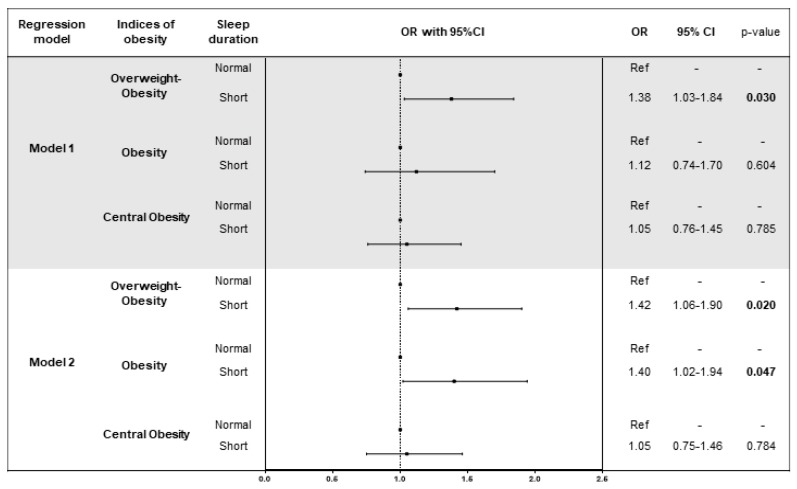
Logistic regression models of the association between sleep duration and indices of obesity. Model 1 was adjusted for age, sex, smoking status, marital status, employment, risk of sleep apnoea, medical history and treatments (diabetes, hypertension and hyperlipidaemia). Model 2 was adjusted for age, sex, smoking status, marital status, employment, medical history and treatments (diabetes, hypertension and hyperlipidaemia), diet, total energy intake and physical activity.

After adjusting for age, sex, neck circumference, smoking status, marital status, employment, risk of sleep apnoea, medical history and treatments of diseases (diabetes, hypertension and hyperlipidaemia), we found that short sleep duration was associated with greater odds of overweight-obesity (BMI > 25) (OR = 1.38; 95%CI = 1.03–1.84; *p* = 0.030) (Figure 1). This finding persisted by adding the effect of diet and physical activity to the model (Model 2; OR = 1.42; 95%CI = 1.06–1.90; *p* = 0.020). In addition, we found that short sleep duration was associated with greater odds of obesity (BMI > 30) in Model 2 (OR = 1.40; 95%CI = 1.02–1.94; *p* = 0.047). Importantly, when we investigated the interactions between sleep duration and EDS, diet and physical activity on BMI and WHR, we did not observe any interaction (*p*-values > 0.05).

### 3.5. Association of Daytime Sleepiness with Indices of Obesity 

**Figure 2:**
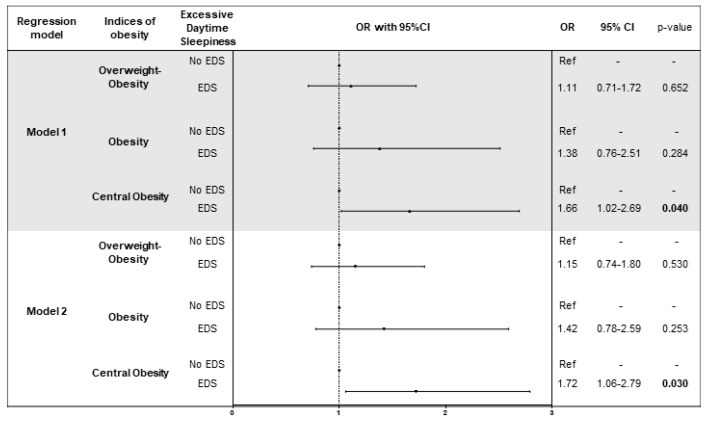
Logistic regression models of the association between excessive daytime sleepiness and indices of obesity. Model 1 was adjusted for age, sex, smoking status, marital status, employment, risk of sleep apnoea, medical history and treatments (diabetes, hypertension and hyperlipidaemia). Model 2 was adjusted for age, sex, smoking status, marital status, employment, medical history and treatments (diabetes, hypertension and hyperlipidaemia), diet, total energy intake and physical activity.

After adjusting for age, sex, neck circumference, smoking status, marital status, employment, risk of sleep apnoea, medical history and treatments of diseases (diabetes, hypertension and hyperlipidaemia), we found that EDS was associated with greater odds of central obesity than no EDS (OR = 1.66; 95%CI = 1.02–2.69; *p* = 0.040) (Figure 2). Interestingly, this finding persisted by adding to the model the effect of diet and physical activity (OR = 1.72; 95%CI = 1.06–2.79; *p* = 0.030) and the association was similar among normal (OR = 1.93; 95%CI = 1.02–3.81; *p* = 0.046) and short sleepers (OR = 1.92; 95%CI = 1.01–4.09; *p* = 0.042). As we noted for sleep duration, we did not observe an interaction between EDS, diet and physical activity (*p*-values > 0.05).

## 4. Discussion

In the present study, we investigated the association between self-reported sleep characteristics and indices of obesity and demonstrated that short sleep duration and EDS are associated with obesity independently of diet and physical activity. Nocturnal sleep duration and EDS have a profound impact on human well-being at multiple levels, increasing all-cause mortality [4,32]. Although adults need ≥7 h of sleep per night for optimal health and wellbeing, it is concerning that in the last two decades, sleep duration has decreased by >1 h in many countries [33,34]. A typical U-shaped association between sleep duration and adverse outcomes of clinical relevance has been reported [32]. As such, our findings, together with those previously reported, raise the need of novel strategies to restore sleep duration into the healthy range of 7–9 h per night, tackling the obesity pandemic [24]. The global increase in the prevalence of obesity is due, in part, to changes in lifestyle and environmental factors, such as dietary habits, physical activity and sedentary behaviour. Although sleep hygiene recommendations include both behavioural and environmental practices helping promote sleep duration and quality [35], further research is needed to explore the interaction with lifestyle factors.

In our Czech cohort, the number of subjects who sleep >9 h was <1% and ~30% sleep <7 h per night; a similar pattern has been observed in US cohorts [36]. Importantly, we demonstrate the association of short sleep duration and EDS with obesity and central obesity. These results are in line with evidence relying on self-reported data [8], which have been recently confirmed also using objective sleep measurement devices (wristbands) [37]. Although previous studies demonstrated the independent association between short sleep duration and obesity after adjusting for diet and physical activity [13,15], our study is the first to examine this relationship in a region where epidemiological studies are scarce, focusing on a population with peculiar lifestyles. Czech people perform very poorly as of metabolic and cardiovascular health, ranking among the top in Europe for incidence of obesity and for the number of lethal events due to CVD [21,22]. Accordingly, while more than half of Kardiovize subjects are very physically active, only 3.9% eat at least four healthy dietary components defined by the American Heart Association (AHA) [31]. With this in mind, the observed lack of association between sleep characteristics, physical activity and diet is surprising in this study. Large epidemiological data linking sleep duration with energy expenditure are inconclusive and indicate a non-linear relationship, as highlighted by a recent AHA scientific statement [38]. Interestingly, in line with recent findings of the UK National Diet and Nutrition Survey [39], we observed that poor dietary habits did not affect the association between irregular sleep patterns and indices of obesity. However, a previous study, demonstrating a positive association between adequate sleep duration and high adherence to the Mediterranean diet [40], suggests that differences in the interaction between sleep and dietary habits could be explained by the geographical diversities of dietary patterns.

EDS is commonly assumed to be the consequence of inadequate sleep duration and/or sleep apnoea [41]. However, emerging evidence indicates several symptoms that are associated with EDS beyond sleep disturbance, suggesting that the mechanism of EDS is multifactorial [41]. Previous cross-sectional studies have reported an association between EDS and obesity measured in terms of BMI [41,42] and others specifically demonstrated this relationship in the absence of sleep-disordered breathing [43,44]. By contrast, a recent longitudinal study showed that EDS was not a significant predictor of the incidence of obesity [45]. Obstructive sleep apnoea—a sleep disorder characterized by repetitive episodes of upper airway obstruction—appears to form a vicious cycle with obesity where each results in worsening of the other [46]. In fact, EDS is related to the increased incidence of obstructive sleep apnoea in obese patients [47], compromising their quality of life. Patients suffering from EDS also experience impairments to their work performance, interpersonal relations and cognitive functions and are at higher risk of causing vehicle accidents and developing metabolic and cardiovascular disturbances [48]. To rule out the effect of sleep apnoea in mediating the link between EDS and obesity, we excluded patients diagnosed with obstructive sleep apnoea from our analysis. While we failed in confirming the relationship between EDS and obesity, we demonstrated an association with central obesity. Notably, we noted for the first time that this relationship persisted after adjusting for diet, physical activity and features related to sleep apnoea.

Several possible mechanisms might underlie the independent association between sleep duration, EDS and obesity. Although the field is characterized by mixed results, sleep curtailment is believed to induce metabolic and endocrine alterations, by decreasing glucose tolerance and insulin sensitivity, increasing evening concentrations of cortisol, increasing levels of ghrelin, decreasing levels of leptin and increasing hunger and appetite [49]. In the context of sleep restriction, it has been observed also an up-regulation of reward, pleasure and salience humoral networks in response to food stimuli. These would predispose one to choose high fat, high carbohydrate foods over balanced meals [49]. Moreover, a study conducted on 19 healthy young men showed that sleep restriction results in elevations in nocturnal and early-morning fatty acid levels accompanied by marked alterations in hormones involved in lipolysis and reduced insulin sensitivity [50].

There are some limitations to our study. The cross-sectional design does not rule out causality but several studies have assessed the impact of sleep restriction on change in body weight [51,52,53]. By contrast, it is possible that strategies aimed at losing weight or reducing waist circumference, might improve EDS by decreasing obstructive sleep apnoea severity [54]. We recognized that self-reported assessment of weekday sleep duration and sleep disorders restricted the power of our work, while, in the era of the big data, the widespread availability and acceptance of electronic wearable devices may allow for more accurate and reliable objective sleep assessment. However, we used validated tools (i.e., Epworth Sleepiness Scale and Berlin questionnaire) and standardized protocol to test our hypothesis. Moreover, sleep duration is a dynamic biological process regulated by the circadian system [55,56] and single measure may not fully capture the sustained effects of sleep duration over time. Finally, although we adjusted for several variables we cannot rule out the possibility of bias from residual confounders that might affect both sleep habits and the risk of obesity, such as light exposure, alcohol consumption, meal timing and other unmeasured socio-demographic factors.

## 5. Conclusions

In summary, we have confirmed a cross-sectional relationship between sleep duration, daytime sleepiness and indices of obesity. Independent of other lifestyle factors, sleeping <7 h increases the risk of overweight-obesity, while EDS seems to be associated with central obesity. These findings suggest that sleep hygiene - the strongest remedy to increase sleep duration into the healthy range of 7–9 h per night—could increase the quality of sleep, prevent EDS and may help prevent or reduce overweight and central obesity, independent of other risk factors. However, strategies aimed to lose weight might in turn affect sleep duration and EDS, therefore, further prospective large-scale studies are needed to evaluate the etiological link and causality between sleep disturbances and obesity.

## Figures and Tables

**Table 1:** Characteristics of study participants by categories of sleep duration.

Characteristics	Sleep Duration			*p*-Value ^b^
	Short (*n* = 457)	Normal (*n* = 1011)	Long (*n* = 14)	
Age, years	48.0 (22.0)	44.5 (20.0)	29.0 (20.0)	<0.001
Sex (% male)	48.4%	51.7%	28.6%	0.126
Marital status (% married)	65.4%	63.9%	40.0%	0.247
Employment (% workers)	80.7%	83.1%	77.8%	0.567
Smoking status (% current)	21.4%	19.9%	20.0%	0.838
Diet				
0–1 components	11.4%	14.3%	11.3%	0.131
2–3 components	84.2%	82.4%	80.3%	
4–5 components	4.4%	3.4%	8.5%	
Total energy intake, Kcal ^a^	2077 (966)	2063 (968)	1925 (755)	0.311
Physical activity, MET-min/week ^a^	3876 (5400)	3035 (4352)	1896 (3774)	<0.001
Physical activity categories				
Low	11.8%	14.2%	7.1%	0.219
Moderate	29.5%	32.0%	50.0%	
High	58.6%	53.7%	42.9%	
Diabetes	9.8%	6.8%	0%	0.073
Use of antidiabetics	5.2%	2.5%	0.0%	0.051
Hypertension	42.0%	35.6%	28.6%	0.052
Use of antihypertensives	21.9%	16.1%	20.0%	0.063
Hyperlipidaemia	64.7%	68.1%	78.6%	0.289
Use of hypolipidaemics	8.9%	6.2%	10.0%	0.235
ESS ^a^	6.0 (6.0)	5.0 (4.0)	3.0 (4.0)	<0.001
High risk of sleep apnoea	27.1%	17.4%	14.3%	<0.001

^a^ Data reported as median (IQR), ^b^ *p*-value based on the Kruskal–Wallis test for continuous variables or the Chi-square test for categorical variables. Abbreviations: IQR, interquartile range; ESS, Epworth Sleepiness Scale.

**Table 2:** Anthropometric measures and indices of obesity by categories of sleep duration.

Characteristics	Sleep Duration			*p*-Value ^b^
	Short (*n* = 457)	Normal (*n* = 1011)	Long (*n* = 14)	
BMI, Kg/m^2 a^	26.7 (6.3)	24.7 (5.4)	23.7 (8.3)	lt;0.001
BMI categories				
Underweight	1.8%	2.4%	7.1%	0.002
Normal weight	39.4%	49.4%	57.1%	
Overweight	37.9%	34.1%	21.4%	
Obesity	21.0%	14.1%	14.3%	
WHR ^a^	0.89 (0.16)	0.86 (0.14)	0.78 (0.20)	0.018
Central obesity	45.2%	38.4%	28.6%	0.032
Body fat mass, Kg ^a^	20.0 (13.6)	17.3 (11.6)	17.9 (9.6)	0.004

^a^ Data reported as median (IQR), ^b^ *p*-value based on the Kruskal–Wallis test for continuous variables or the Chi-square test for categorical variables. Abbreviations: IQR, interquartile range; WHR, Waist-Hip Ratio.

**Table 3:** Characteristics of study population by excessive daytime sleepiness.

Characteristics	Excessive Daytime Sleepiness		*p*-Value ^b^
	No EDS (*n* = 1319)	EDS (*n* = 163)	
Age, years	44.0 (20.0)	48.5 (22.0)	0.711
Sex (% male)	49.6%	58.3%	0.036
Marital status (% married)	63.5%	72.3%	0.058
Employment (% workers)	81.9%	87.4%	0.136
Smoking status (% current)	21.1%	14.3%	0.081
Diet			
0–1 components	13.2%	13.5%	0.836
2–3 components	82.7%	83.4%	
4–5 components	4.0%	3.1%	
Total energy intake, Kcal ^a^	2049 (948)	2136 (888)	0.002
Physical activity, MET-min/week ^a^	3074 (4164)	2253 (5773)	0.406
Physical activity categories			
Low	13.5%	13.5%	0.832
Moderate	31.7%	29.4%	
High	54.8%	57.1%	
Diabetes	7.8%	6.7%	0.625
Use of antidiabetics	3.3%	3.4%	0.957
Hypertension	37.0%	41.7%	0.239
Use of antihypertensives	18.1%	16.0%	0.569
Hyperlipidaemia	66.9%	69.3%	0.538
Use of hypolipidaemics	7.3%	5.0%	0.361
Short sleep duration	28.6%	46.6%	<0.001
High risk of sleep apnoea	19.5%	27.0%	0.026

^a^ Data reported as median (IQR), ^b^ *p*-value based on the Mann-Whitney U test for continuous variables or the Chi-square test for categorical variables. Abbreviations: IQR, interquartile range; ESS, Epworth Sleepiness Scale.

**Table 4:** Anthropometric measures and indices of obesity by excessive daytime sleepiness.

Characteristics	Excessive Daytime Sleepiness		*p*-Value ^b^
	No EDS (*n* = 1319)	EDS (*n* = 163)	
BMI, Kg/m^2 a^	25.0 (6.3)	28.6 (7.3)	0.020
BMI categories			
Underweight	2.4%	1.2%	0.150
Normal weight	47.1%	39.9%	
Overweight	34.8%	38.0%	
Obesity	15.7%	20.9%	
WHR ^a^	0.87 (0.14)	0.93 (0.12)	0.013
Central obesity	38.9%	52.1%	0.001
Body fat mass, Kg ^a^	17.9 (12.2)	20.0 (11.9)	0.772
BMI, Kg/m^2 a^	38.0 (7.0)	39.0 (5.0)	0.005

^a^ Data reported as median (IQR), ^b^ *p*-value based on the Mann-Whitney U test for continuous variables or the Chi-square test for categorical variables. Abbreviations: IQR, interquartile range; WHR, Waist-Hip Ratio.
